# Repositioned donafenib versus standard regorafenib for second-line HCC treatment: A matched cohort study

**DOI:** 10.1097/MD.0000000000048470

**Published:** 2026-04-24

**Authors:** Xiaoshuang Chen, Xianyong Li, Xin Li, Yu Jiang, Qingyun Xie, Ping Xie, Lei Cao

**Affiliations:** a Department of Pharmacy, West China Hospital, Sichuan University, Chengdu, Sichuan, People’s Republic of China; b Department of Radiology, Chengdu Xinjin District People’s Hospital, Chengdu, Sichuan, People’s Republic of China; c Department of Interventional Radiology, People’s Hospital of Deyang City, Deyang, Sichuan, People’s Republic of China; d General Surgery Department, Southwest Medical University Affiliated Hospital, Luzhou, Sichuan, People’s Republic of China; e Liver Transplantation Center, State Key Laboratory of Biotherapy and Cancer Center, West China Hospital, Sichuan University and Collaborative Innovation Center of Biotherapy, Chengdu, Sichuan, People’s Republic of China; f Department of Radiology, Sichuan Academy of Medical Sciences, Sichuan Provincial People’s Hospital, University of Electronic Science and Technology of China, Chengdu, Sichuan, People’s Republic of China.

**Keywords:** bevacizumab, donafenib, drug repositioning, hepatocellular carcinoma, immunotherapy, propensity score matching, regorafenib, second-line treatment

## Abstract

The dominance of bevacizumab plus immunotherapy in first-line advanced hepatocellular carcinoma (HCC) has created opportunities for repositioning previously effective agents in treatment sequences. We compared donafenib, a first-line approved agent repositioned for second-line use, versus regorafenib, the established second-line standard, both combined with programmed cell death protein 1 (PD-1) inhibitors (immune checkpoint inhibitors). This retrospective matched cohort study analyzed patients with advanced HCC receiving either donafenib combined with PD-1 inhibitors or regorafenib combined with PD-1 inhibitors as second-line treatment after bevacizumab plus immunotherapy failure (2021–2024). Propensity score matching (1:1) balanced baseline characteristics. Primary endpoints were progression-free survival and overall survival. After matching, 50 patients were included (25 per group). Donafenib combined with PD-1 inhibitors achieved median progression-free survival of 9.3 months versus 7.1 months for regorafenib combined with PD-1 inhibitors (hazard ratio 1.114, 95% confidence interval: 0.581–2.134, *P* = .745). Median overall survival was 25.8 versus 17.4 months, respectively (hazard ratio 1.517, 95% confidence interval: 0.685–3.359, *P* = .304). Objective response rate was 28.0% versus 20.0% (*P* = .742), and disease control rate was 56.0% versus 48.0% (*P* = .778). Treatment-related adverse events occurred in 76.0% versus 84.0% of patients (*P* = .725), with grade 3/4 events in 16.0% versus 24.0%. Donafenib repositioned as second-line therapy demonstrated comparable efficacy and safety to standard regorafenib-based treatment in advanced HCC following bevacizumab plus immunotherapy failure. These findings support strategic repositioning of underutilized first-line agents and provide evidence for expanding second-line treatment options in advanced HCC.

## 1. Introduction

Hepatocellular carcinoma (HCC) ranks sixth in the incidence of malignant tumors and is the fourth leading cause of cancer-related deaths globally, accounting for over 90% of primary liver cancers.^[1]^ The burden of HCC varies considerably across different countries and regions, with approximately 80 to 90% of cases occurring within the context of underlying liver cirrhosis.^[2]^ Despite advances in early diagnosis, most HCC patients are diagnosed at an advanced stage, precluding curative surgical intervention and necessitating systemic therapy as the primary treatment modality.^[3]^

The landscape of first-line systemic treatment for advanced HCC has undergone a paradigm shift in recent years. While tyrosine kinase inhibitors such as sorafenib initially dominated the therapeutic armamentarium,^[4]^ the emergence of immunotherapy combinations has fundamentally altered treatment paradigms. The IMbrave150 trial positioned atezolizumab plus bevacizumab (the “T + A” regimen) as first-line standard care, showing superior overall survival (OS) and progression-free survival (PFS) versus sorafenib.^[5]^ The ORIENT-32 study further validated this approach, with sintilimab plus bevacizumab biosimilar (IBI305) outperforming sorafenib in unresectable or metastatic disease.^[6]^

With bevacizumab-containing immunotherapy regimens now dominating first-line treatment, many previously effective agents face marginalization despite their proven therapeutic value. Several drugs with established first-line efficacy, including sorafenib, lenvatinib, and donafenib, now face significantly reduced utilization rates despite their proven therapeutic value. Donafenib, when compared to sorafenib, demonstrated longer OS in advanced HCC patients with similar median PFS and received approval for treatment of unresectable HCC in China.^[7]^ However, the clinical dominance of the T + A regimen has relegated these agents to secondary consideration, creating an opportunity for their strategic repositioning in the treatment sequence.

Second-line options for advanced HCC are constrained, with regorafenib, cabozantinib, and ramucirumab providing modest clinical benefits.^[8–10]^ Among these, regorafenib was the initial second-line approval, extending OS and PFS in RESORCE trial participants following sorafenib progression.^[8]^ Similarly, cabozantinib demonstrated significant OS extension in the CELESTIAL trial, particularly in patients who had received sorafenib as their only prior systemic treatment.^[10]^ Despite these advances, the clinical need for additional effective second-line options remains substantial, particularly given the heterogeneity of patient responses and tolerance profiles.

The theoretical foundation for immune checkpoint inhibitor (ICI) continuation following first-line immunotherapy failure remains an active area of investigation. The liver’s unique immune microenvironment, characterized by inherent immune tolerance mechanisms, suggests potential for sequential immunotherapy approaches.^[11]^ Anti-angiogenic therapy can suppress tumor microenvironment angiogenesis and immune suppression, potentially enhancing the efficacy of programmed cell death protein 1 (PD-1) and programmed death-ligand 1 inhibitors through complementary mechanisms.^[5]^ These observations support the rationale for combining targeted therapy with immunotherapy in second-line settings, even following initial immunotherapy exposure.

Real-world evidence has become increasingly valuable in guiding clinical decision-making for HCC treatment, particularly given the complexity of patient selection and treatment sequencing. Such evidence can provide insights into treatment effectiveness under routine clinical conditions, complementing the controlled environment findings of randomized clinical trials and offering guidance for individualized treatment strategies in diverse patient populations.^[12]^

The present study addresses a critical gap in the current treatment landscape by directly comparing the efficacy and safety of donafenib versus regorafenib, each combined with ICIs, as second-line therapy in patients with advanced HCC who progressed following first-line bevacizumab plus immunotherapy. To our knowledge, this is the first study directly comparing a repositioned first-line agent with standard second-line therapy in this clinical setting, both in combination with immunotherapy. The findings may provide evidence-based guidance for optimizing treatment sequences and expanding therapeutic options for patients with advanced HCC, potentially offering a framework for the strategic repositioning of underutilized first-line agents in contemporary clinical practice.

## 2. Material and methods

### 2.1. Study population

This study was approved by the Medical Ethics Committee of Sichuan Academy of Medical Sciences & Sichuan Provincial People’s Hospital (No. 2021-40-2), and the requirement for informed consent was waived due to its retrospective nature. We conducted a retrospective analysis of advanced HCC patients receiving treatment at our center from 2021 to 2024. Eligible participants had histologically or radiologically confirmed unresectable or metastatic HCC with documented disease progression following first-line bevacizumab plus ICI therapy. Patients subsequently received either donafenib combined with PD-1 inhibitors (Dona + IO group) or regorafenib combined with PD-1 inhibitors (Rego + IO group) as second-line treatment.

Inclusion criteria encompassed patients aged 18 years or older with Eastern Cooperative Oncology Group performance status of 0 to 2, Child-Pugh class A or B liver function, and adequate organ function parameters. Exclusion criteria included incomplete medical records, loss to follow-up before first disease assessment, concurrent participation in clinical trials involving investigational agents, and previous exposure to either donafenib or regorafenib in any treatment line.

From an initial cohort of 143 patients, 36 were excluded due to incomplete documentation (n = 17) or loss to follow-up (n = 19), leaving 107 evaluable patients for analysis. Following propensity score matching (PSM), 50 patients formed the final analytical cohort.

### 2.2. Treatment procedures

Donafenib was administered orally at 200 mg twice daily on a continuous basis, with dose modifications permitted for toxicity management. Regorafenib was given orally at 160 mg once daily for 21 days of each 28-day cycle, following standard dosing protocols. Both regimens were combined with PD-1 inhibitors administered intravenously every 2 to 3 weeks according to institutional guidelines.

Treatment continued until disease progression, unacceptable toxicity, patient withdrawal, or death. Dose reductions and treatment interruptions were managed according to established protocols, with decisions made by the treating oncologist based on individual patient tolerance and toxicity profiles.

### 2.3. Data collection and follow-up

Patient demographics, disease characteristics, treatment history, and laboratory parameters were extracted from electronic medical records, with baseline assessments conducted within 4 weeks prior to treatment initiation. Follow-up evaluations occurred every 6 to 8 weeks during active treatment, incorporating clinical assessments, laboratory monitoring, and radiological imaging according to Response Evaluation Criteria in Solid Tumors v1.1 criteria. Safety monitoring employed Common Terminology Criteria for Adverse Events (AEs) v5.0 for toxicity grading, with survival follow-up continuing beyond treatment discontinuation through clinic visits, telephone contacts, or medical record review until the established data cutoff date.

### 2.4. Outcomes and assessments

Primary endpoints comprised PFS (time from treatment initiation to radiological progression or death) and OS (time from treatment start to death from any cause). Secondary endpoints included objective response rate (ORR) (complete plus partial responses), disease control rate (complete response, partial response, plus stable disease), and safety profile characterization. Radiological assessments were performed by institutional radiologists according to routine clinical practice, with response duration calculated from first documented response to progression or death.

### 2.5. Sample size determination

Given the retrospective nature of this real-world study, no formal a priori sample size calculation was performed. Instead, the sample size was initially determined by including all eligible patients treated at our center during the study period to maximize representativeness. The final analytical sample size was subsequently adjusted through PSM (1:1 ratio) to ensure balanced baseline characteristics, resulting in a total of 50 patients (25 per group).

### 2.6. Statistical analysis

Statistical analysis data are presented as percentages for categorical variables and mean ± standard deviation or median and interquartile range for continuous variables, depending on the normality of distribution. Baseline characteristics were compared using chi-square or Fisher exact tests for categorical variables and independent *t* tests or Mann-Whitney U tests for continuous variables based on distribution normality. PSM employed a 1:1 nearest-neighbor algorithm without replacement, incorporating age, barcelona clinic liver cancer (BCLC) staging, and Child-Pugh classification as primary matching variables. Match quality was assessed using standardized mean differences below 0.1.

Survival analyses utilized Kaplan–Meier methodology with log-rank tests for group comparisons, while hazard ratios (HRs) and 95% confidence intervals (CIs) were estimated using Cox proportional hazards models. Subgroup analyses explored treatment effects across patient characteristics using forest plot visualization. Statistical significance was set at *P* < .05. Analyses were performed using R statistical software version 4.0 (R Foundation for Statistical Computing, Vienna, Austria). Specifically, the “MatchIt” package was used for PSM, the “survival” package was used for survival analysis, and the “ggplot2” package was used for data visualization.

## 3. Results

### 3.1. Patient and tumor characteristics

**Figure 1.**
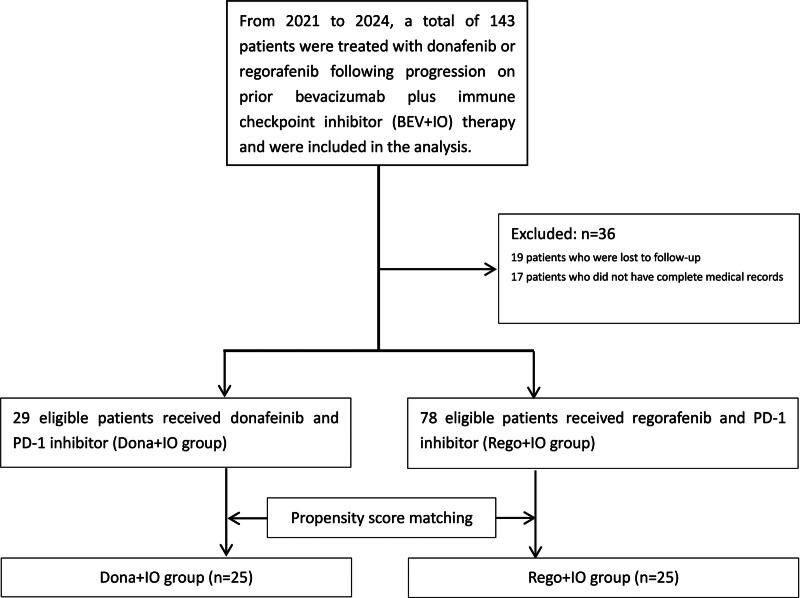
Patient flow chart. From the initial cohort of 143 patients who received donafenib or regorafenib plus PD-1 inhibitor after progression on prior BEV + IO therapy, 36 patients were excluded (19 were lost to follow-up and 17 had incomplete medical records). The remaining 107 eligible patients were allocated into 2 treatment groups: the Dona + IO group (n = 29) and the Rego + IO group (n = 78). Subsequently, propensity score matching was performed at a 1:1 ratio, resulting in 25 well-matched pairs of patients for comparative analysis. BEV + IO = bevacizumab plus immunotherapy, Dona + IO group = donafenib combined with PD‐1 inhibitors, n = number of patients, PD-1 = programmed cell death protein 1, Rego + IO group = regorafenib combined with PD‐1 inhibitors.

During 2021 to 2024, 143 patients received second-line donafenib or regorafenib with immunotherapy after bevacizumab-based progression (Fig. 1). We excluded 36 patients (17 incompleterecords, 19 lost to follow-up), leaving 107 evaluable cases. PSM (1:1) using age, BCLC stage, and Child-Pugh class yielded 50 patients for analysis (25 per arm).

Baseline characteristics were well-balanced between groups (Table 1). Median age was 53.96 ± 12.13 years versus 56.2 ± 11.94 years in Dona + IO and Rego + IO groups (*P* = .5137). Male predominance was observed in both groups (88.0% vs 84.0%, *P* = 1.0000). Hepatitis C infection occurred in 100.0% versus 88.0% of patients (*P* = .2347). BCLC stage C disease was present in 72.0% versus 76.0% of patients (*P* = 1.0000), while Child-Pugh class A function was documented in 68.0% versus 64.0% (*P* = 1.0000). Portal vein tumor thrombosis occurred in 64.0% versus 76.0% of patients (*P* = .5371), and extrahepatic metastasis in 76.0% versus 68.0% (*P* = .7528). Eastern Cooperative Oncology Group performance status 0 was present in 84.0% versus 80.0% of patients (*P* = 1.0000), with elevated Alpha-fetoprotein ( > 400 ng/mL) in 48.0% versus 52.0% (*P* = 1.0000). Laboratory parameters showed no significant differences between groups.

**Table 1:** Baseline characteristics of study participants.

Characteristic	Dona + IO group (n = 25)	Rego + IO group (n = 25)	*P* value
Demographics			
Age, years	53.96 ± 12.13	56.2 ± 11.94	.5137
Male gender	22 (88.0)	21 (84.0)	1.0000
Viral etiology			
HBV positive	4 (16.0)	6 (24.0)	.7237
HCV positive	25 (100.0)	22 (88.0)	.2347
Tumor staging and liver function			
BCLC staging			1.0000
Stage B	7 (28.0)	6 (24.0)	
Stage C	18 (72.0)	19 (76.0)	
Child-Pugh Score			1.0000
Class A	17 (68.0)	16 (64.0)	
Class B	6 (24.0)	8 (32.0)	
Class C	2 (8.0)	1 (4.0)	
Ascites present	16 (64.0)	20 (80.0)	.3447
Tumor characteristics			
Tumor size > 5 cm	12 (48.0)	6 (24.0)	.1407
Tumor number			.4569
Single	10 (40.0)	6 (24.0)	
2–3 lesions	5 (20.0)	7 (28.0)	
≥4 lesions	10 (40.0)	12 (48.0)	
Tumor morphology			.7771
Mass-forming	14 (56.0)	12 (48.0)	
Nodular	7 (28.0)	10 (25.0)	
Diffuse infiltrative	4 (16.0)	3 (27.0)	
PVTT present	16 (64.0)	19 (76.0)	.5371
Extrahepatic metastasis	19 (76.0)	17 (68.0)	.7528
Performance status and biomarkers			
ECOG score 0	21 (84.0)	20 (80.0)	1.0000
AFP > 400 ng/mL	12 (48.0)	13 (52.0)	1.0000
Previous systemic treatments			
Bevacizumab plus immunotherapy	25 (100.0)	25 (100.0)	1.0000
Laboratory parameters			
Hemoglobin, g/L	136 (122–146)	137 (125–151)	.6002
Red blood cell count, ×10^12^/L	4.36 (4.04–4.63)	4.54 (4.02–4.78)	.6979
White blood cell count, ×10^9^/L	5.96 ± 2.6	5.8 ± 1.95	.8069
Neutrophil count, ×10^9^/L	3.77 (2.51–5.29)	3.45 (3.08–4.51)	.8084
Lymphocyte count, ×10^9^/L	0.9 (0.66–1.37)	1.04 (0.83–1.57)	.1032
Platelet count, ×10^9^/L	108 (86–131)	109 (73–143)	.8461
Total bilirubin, μmol/L	18.8 (13.8–30.9)	16.8 (11–21)	.2482
Direct bilirubin, μmol/L	8.8 (4.3–12.1)	6.4 (4.3–10.6)	.3932
ALT, U/L	51 (33–117)	49.5 (29–71)	.8537
AST, U/L	45 (35–138)	48 (31–77)	.5936
Albumin, g/L	37.6 (33.7–42.6)	39.4 (36.98–43.8)	.1566
Prothrombin time, s	13.15 ± 1.72	12.64 ± 1.36	.2529
APTT, s	29.9 (26.7–33.7)	29.7 (28–31)	.9613

AFP = alpha-fetoprotein, ALT = alanine aminotransferase, APTT = activated partial thromboplastin time, AST = aspartate aminotransferase, BCLC = Barcelona Clinic Liver Cancer, Dona + IO group = donafenib combined with PD‐ 1 inhibitors, ECOG = Eastern Cooperative Oncology Group, HBV = hepatitis B virus, HCV = hepatitis C virus, n = number of participants, PD-1 = programmed cell death protein 1, PVTT = portal vein tumor thrombosis, Rego + IO group = regorafenib combined with PD‐1 inhibitors.

### 3.2. Efficacy outcomes

**Figure 2.**
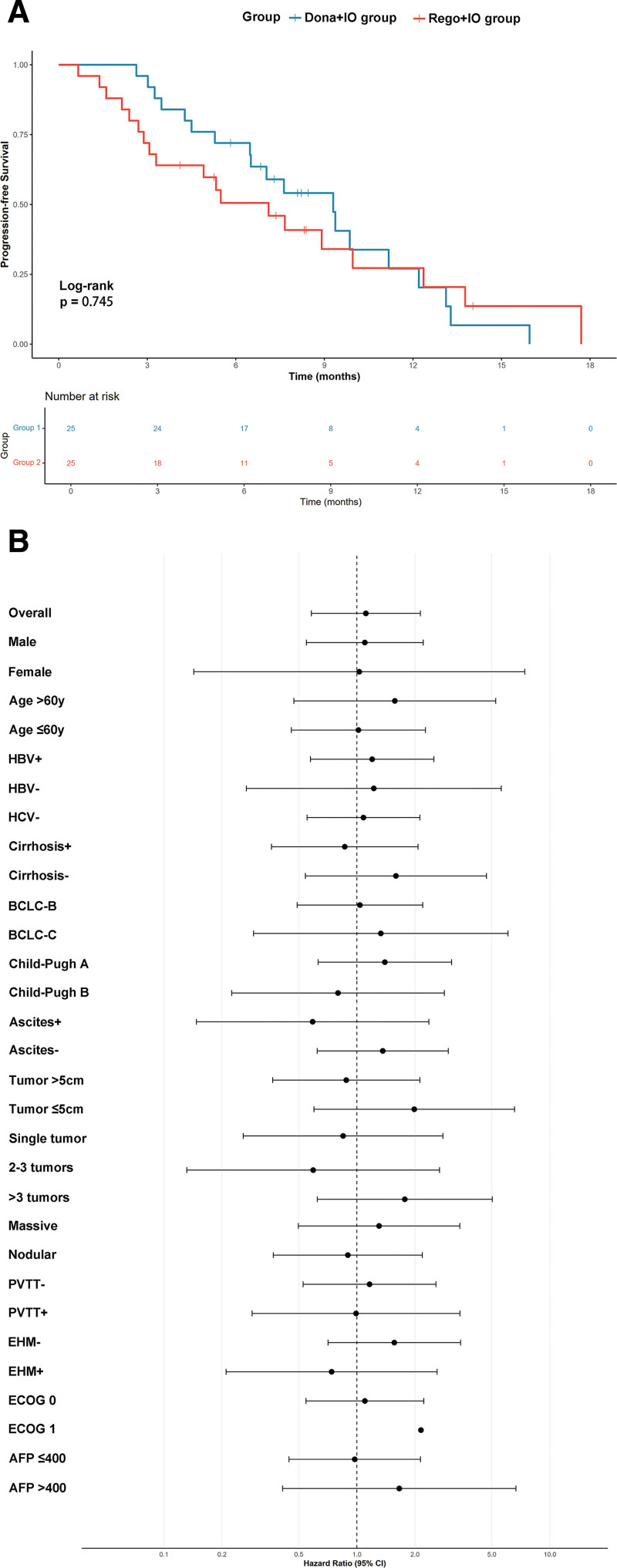
Progression-free survival outcomes. (A) Kaplan–Meier survival curves comparing progression-free survival between the Donafenib + immunotherapy group and Regorafenib + immunotherapy group. The median progression-free survival was 7.1 months (95% CI: 3.3–13.8) in the Regorafenib + immunotherapy group versus 9.3 months (95% CI: 6.5–13.1) in the Donafenib + immunotherapy group (HR 1.114, 95% CI: 0.581–2.134, *P* = .745). (B) Forest plot showing subgroup analysis of progression-free survival across different patient characteristics and clinical variables. HRs and 95% CIs are displayed for each subgroup, with most subgroups showing consistent treatment effects between the 2 approaches. AFP = alpha-fetoprotein, BCLC = Barcelona Clinic Liver Cancer, CI = confidence interval, Dona + IO group = donafenib combined with PD‐1 inhibitors, ECOG = Eastern Cooperative Oncology Group, EHM = extrahepatic metastasis, HBV = hepatitis B virus, HCV = hepatitis C virus, HR = hazard ratio, PD-1 = programmed cell death protein 1, PVTT = portal vein tumor thrombosis, Rego + IO group = regorafenib combined with PD‐1 inhibitors.

At a median follow-up of 15.2 months, donafenib plus immunotherapy demonstrated comparable efficacy to regorafenib plus immunotherapy across primary and secondary endpoints. Median PFS was 7.1 months (95% CI: 3.3–13.8) in the Rego + IO group versus 9.3 months (95% CI: 6.5–13.1) in the Dona + IO group (HR 1.114, 95% CI: 0.581–2.134, *P* = .745); (Fig. 2A). Subgroup analyses demonstrated consistent treatment effects across patient characteristics, with HRs favoring neither treatment approach significantly (Fig. 2B).

**Figure 3.**
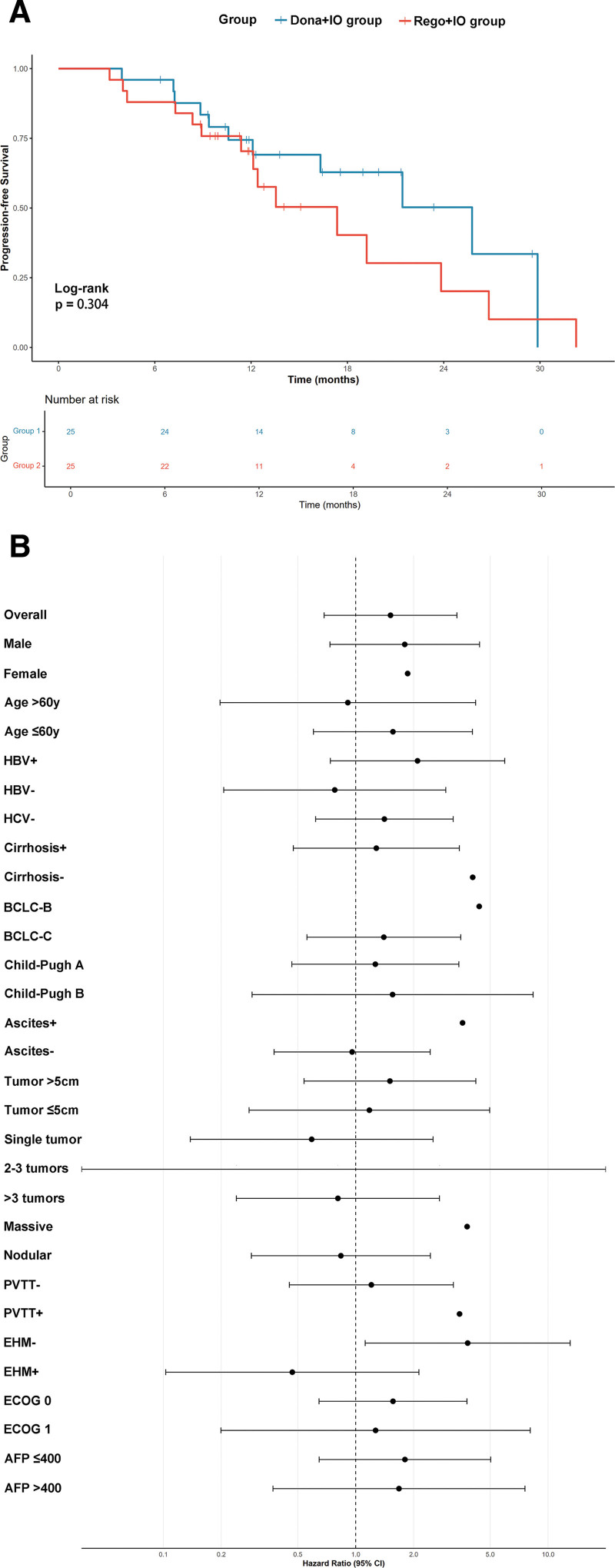
Overall survival outcomes. (A) Kaplan–Meier survival curves comparing Overall Survival between the Donafenib + immunotherapy group and Regorafenib + immunotherapy group. The median Overall Survival was 17.4 months (95% CI: not reached) in the Regorafenib + immunotherapy group versus 25.8 months (95% CI: not reached) in the Donafenib + immunotherapy group (HR 1.517, 95% CI: 0.685–3.359, *P* = .304). (B) Forest plot showing subgroup analysis of Overall Survival across different patient characteristics and clinical variables. Hazard ratios and 95% confidence intervals are displayed for each subgroup, with most subgroups showing consistent treatment effects between the 2 approaches. AFP = alpha-fetoprotein, BCLC = Barcelona Clinic Liver Cancer, CI = confidence interval, Dona + IO group = donafenib combined with PD‐1 inhibitors, ECOG = Eastern Cooperative Oncology Group, EHM = extrahepatic metastasis, HBV = hepatitis B virus, HCV = hepatitis C virus, HR = hazard ratio, PD-1 = programmed cell death protein 1, PVTT = portal vein tumor thrombosis, Rego + IO group = regorafenib combined with PD‐1 inhibitors.

OS outcomes showed a median survival of 17.4 months (95% CI: not reached) in the Rego + IO group compared with 25.8 months (95% CI: not reached) in the Dona + IO group (HR 1.517, 95% CI: 0.685–3.359, *P* = .304); (Fig. 3A). Subgroup analyses for OS similarly demonstrated consistent treatment effects across predefined patient characteristics (Fig. 3B).

ORRs were comparable between treatment groups (Table 2). Complete response was achieved in 1 patient (4.0%) in the Dona + IO group versus none in the Rego + IO group. Partial responses occurred in 24.0% (6/25) and 20.0% (5/25) of patients, respectively, resulting in ORRs of 28.0% versus 20.0% (*P* = .742). Stable disease was documented in 28.0% (7/25) of patients in both groups, while progressive disease occurred in 44.0% (11/25) versus 52.0% (13/25) of Dona + IO and Rego + IO patients, respectively. Disease control rates, defined as complete response plus partial response plus stable disease, were 56.0% (14/25) in the Dona + IO group and 48.0% (12/25) in the Rego + IO group (*P* = .778).

**Table 2:** Comparison of best overall response and efficacy outcomes between 2 groups.

Variable	Dona + IO group (n = 25)	Rego + IO group (n = 25)	*P* value
Best overall response, n (%)			
CR	1 (4.0)	0 (0.0)	
PR	6 (24.0)	5 (20.0)	
SD	7 (28.0)	7 (28.0)	
PD	11 (44.0)	13 (52.0)	
ORR, n (%)	7 (28.0)	5 (20.0)	.742
DCR, n (%)	14 (56.0)	12 (48.0)	.778

CR = Complete Response, DCR = Disease Control Rate (CR + PR + SD), Dona + IO group = donafenib combined with PD‐1 inhibitors, n = number of participants, ORR = Objective Response Rate (CR + PR), PD-1 = programmed cell death protein 1, PD = Progressive Disease, PR = Partial Response, Rego + IO group = regorafenib combined with PD‐ 1 inhibitors, SD = Stable Disease.

*P* values calculated using Chi-square test or Fisher exact test as appropriate.

### 3.3. Prognostic factor analysis

Comprehensive subgroup analyses evaluated treatment effects across predefined patient characteristics for both PFS and OS. For PFS, subgroup analyses revealed consistent treatment patterns across demographic and clinical variables (Table S1, Supplemental Digital Content, https://links.lww.com/MD/R749). Gender-based analysis showed similar outcomes in male patients (HR 1.100, 95% CI: 0.548–2.209, *P* = .789) and females (HR 1.029, 95% CI: 0.143–7.417, *P* = .977). Age stratification demonstrated comparable effects in patients ≤ 60 years (HR 1.019, 95% CI: 0.458–2.267, *P* = .964) versus > 60 years (HR 1.573, 95% CI: 0.472–5.242, *P* = .461). Disease staging showed uniform effects across BCLC stages, with stage B patients demonstrating HR 1.331 (95% CI: 0.292–6.072, *P* = .712) and stage C patients HR 1.038 (95% CI: 0.491–2.195, *P* = .923).

OS subgroup analyses demonstrated similar consistency (Table S2, Supplemental Digital Content, https://links.lww.com/MD/R749). A notable finding emerged in patients without extrahepatic metastasis (HR 3.820, 95% CI: 1.120–13.025, *P* = .032), though this represented a small subgroup requiring cautious interpretation. Most predefined subgroups demonstrated HRs crossing unity with wide CIs, indicating no clear advantage for either treatment approach across diverse patient populations and supporting the overall conclusions of comparable efficacy between treatment regimens.

### 3.4. Safety and tolerability

Both treatment regimens demonstrated manageable safety profiles with comparable toxicity patterns (Table 3). Treatment-related AEs of any grade occurred in 76.0% (19/25) of Dona + IO patients versus 84.0% (21/25) of Rego + IO patients (*P* = .725). Grade 3/4 AEs were less frequent in the Dona + IO group at 16.0% (4/25) compared with 24.0% (6/25) in the Rego + IO group.

**Table 3:** Treatment-related adverse events between 2 groups.

Event, n (%)	Dona + IO group (n = 25)			Rego + IO group (n = 25)			*P* value
	Any grade	Grade 1/2	Grade 3/4	Any grade	Grade 1/2	Grade 3/4	Any grade
Any TRAE	19 (76.0)	15 (60.0)	4 (16.0)	21 (84.0)	15 (60.0)	6 (24.0)	.725
Hand and foot syndrome	4 (16.0)	3 (12.0)	1 (4.0)	5 (20.0)	4 (16.0)	1 (4.0)	1.000
Increased AST	2 (8.0)	1 (4.0)	1 (4.0)	3 (12.0)	2 (8.0)	1 (4.0)	1.000
Increased ALT	2 (8.0)	2 (4.0)	0 (0.0)	2 (14.0)	1 (4.0)	1 (4.0)	1.000
Fatigue	1 (4.0)	1 (4.0)	0 (0.0)	1 (4.0)	1 (4.0)	0 (0.0)	1.000
Fever	1 (4.0)	1 (4.0)	0 (0.0)	1 (4.0)	1 (4.0)	0 (0.0)	1.000
Rash	1 (4.0)	1 (4.0)	0 (0.0)	1 (4.0)	1 (4.0)	0 (0.0)	1.000
Increased blood bilirubin	1 (4.0)	1 (4.0)	0 (0.0)	1 (4.0)	1 (4.0)	0 (0.0)	1.000
Thrombocytopenia	2 (8.0)	1 (4.0)	1 (4.0)	1 (4.0)	1 (4.0)	0 (0.0)	1.000
Gastrointestinal hemorrhage	1 (4.0)	0 (0.0)	1 (4.0)	0 (0.0)	0 (0.0)	0 (0.0)	1.000
Decreased appetite	0 (0.0)	0 (0.0)	0 (0.0)	1 (4.0)	0 (0.0)	1 (0.0)	1.000
Diarrhea	1 (4.0)	1 (4.0)	0 (0.0)	1 (4.0)	0 (0.0)	1 (4.0)	1.000
Hypertension	0 (0.0)	0 (0.0)	0 (0.0)	1 (4.0)	1 (4.0)	0 (0.0)	1.000
Pruritus	1 (4.0)	1 (4.0)	0 (0.0)	1 (4.0)	1 (4.0)	0 (0.0)	1.000
Proteinuria	1 (4.0)	1 (4.0)	0 (0.0)	1 (4.0)	1 (4.0)	0 (0.0)	1.000
Leukopenia	0 (0.0)	0 (2.0)	0 (0.0)	1 (4.0)	0 (0.0)	1 (0.0)	1.000
Gingival bleeding	1 (4.0)	1 (0.0)	0 (0.0)	0 (0.0)	0 (0.0)	0 (0.0)	1.000

ALT = alanine aminotransferase, AST = aspartate aminotransferase, Dona + IO group = donafenib combined with PD‐1 inhibitors, n = number of participants, PD-1 = programmed cell death protein 1, Rego + IO group = regorafenib combined with PD‐ 1 inhibitors, TRAE = Treatment-Related Adverse Event.

*P* values calculated using Fisher exact test. Only events occurring in ≥ 1 patient in either group are shown. All adverse events were graded according to Common Terminology Criteria for Adverse Events (CTCAE) version 5.0.

The most common AE was hand-foot syndrome, occurring in 16.0% versus 20.0% of patients (*P* = 1.000). Hepatotoxicity manifested as increased aspartate aminotransferase in 8.0% versus 12.0% of patients and increased alanine aminotransferase in 8.0% versus 14.0% of patients, respectively. Thrombocytopenia occurred in 8.0% of Dona + IO patients versus 4.0% of Rego + IO patients. Constitutional symptoms including fatigue, fever, and rash were infrequent, each affecting 4.0% of patients in both groups. Gastrointestinal events included 1 case of hemorrhage in the Dona + IO group and 1 case of decreased appetite in the Rego + IO group. No treatment-related deaths occurred in either group.

## 4. Discussion

This propensity score-matched analysis represents the first direct comparative study of donafenib repositioned as second-line therapy versus standard regorafenib-based treatment in patients with advanced HCC following progression on bevacizumab plus immunotherapy. Both treatment strategies showed similar effectiveness and tolerability, offering valuable real-world insights for clinical practice in the evolving landscape of HCC management.

Advanced HCC treatment paradigms have shifted dramatically as bevacizumab-immunotherapy combinations gained first-line prominence. The IMbrave150 trial established atezolizumab plus bevacizumab as the new gold standard, demonstrating superior OS and PFS compared to sorafenib,^[5]^ while the ORIENT-32 trial further validated sintilimab plus bevacizumab biosimilar for first-line treatment.^[6]^ This paradigm shift has created an unprecedented clinical scenario where multiple previously effective first-line agents, including sorafenib, lenvatinib, and donafenib, now face significantly reduced utilization despite their established therapeutic value.^[4,7,13,14]^ The Chinese Guidelines for Diagnosis and Treatment of Primary Liver Cancer (2024 edition) acknowledge this evolving landscape, highlighting the challenges of treatment selection in the era of expanding first-line options.^[3]^ However, the expanding arsenal of first-line options contrasts sharply with the limited evidence-based second-line treatment recommendations, with regorafenib remaining one of the few standard options supported by phase III trial data.^[8]^

Our study directly addresses this critical knowledge gap by examining the repositioning of first-line agents to second-line settings. The median PFS of 9.3 months observed with donafenib plus immunotherapy compares favorably with historical second-line data. In the RESORCE study, regorafenib monotherapy achieved a median PFS of 3.1 months versus 1.5 months with placebo in patients progressing on sorafenib.^[8]^ Similarly, cabozantinib demonstrated a median PFS of 5.2 months in the CELESTIAL trial,^[10]^ while ramucirumab showed 2.8 months in Alpha-fetoprotein -elevated patients in REACH-2^9^. The enhanced performance in our study likely reflects the synergistic mechanisms between anti-angiogenic agents and immunotherapy, consistent with preclinical evidence demonstrating that anti-angiogenic therapy can suppress tumor microenvironment angiogenesis and immune suppression.^[5,11]^

The OS advantage observed numerically with donafenib plus immunotherapy (25.8 months vs 17.4 months) warrants careful interpretation despite the lack of statistical significance. This 8.4-month difference represents a clinically meaningful survival benefit that aligns with donafenib’s distinct pharmacological properties. In the pivotal phase III trial comparing donafenib to sorafenib, donafenib demonstrated longer OS in advanced HCC patients with similar median PFS,^[7]^ suggesting unique antitumor mechanisms that may be preserved when repositioned as second-line therapy.^[15,16]^

The rationale for continuing immunotherapy following progression on first-line bevacizumab-based combinations remains an active area of investigation. The liver’s unique immune microenvironment, characterized by inherent immune tolerance mechanisms, supports the potential for sequential immunotherapy approaches.^[11,17]^ Additionally, anti-angiogenic therapy can enhance the efficacy of PD-1/programmed death-ligand 1 inhibitors through complementary mechanisms involving vascular normalization and improved immune cell infiltration.^[5]^ Current expert recommendations acknowledge this theoretical foundation, suggesting consideration of agents with different mechanisms of action following progression on bevacizumab-based combinations.^[18–20]^

The safety profiles observed in both treatment arms align with known toxicity patterns from previous studies, with no unexpected AEs identified. The comparable incidence of treatment-related AEs (76.0% versus 84.0%) and grade 3/4 toxicities (16.0% vs 24.0%) support the feasibility of both regimens in the second-line setting.^[21,22]^ These findings are particularly relevant given the limited safety data for continuing immunotherapy after progression on first-line bevacizumab-based combinations.^[23]^

Several limitations warrant consideration in interpreting our findings. The retrospective design introduces inherent selection bias despite PSM efforts to balance key prognostic factors. The relatively small sample size of 50 patients limits statistical power to detect meaningful differences between groups, particularly for OS endpoints. Additionally, the heterogeneity in PD-1 inhibitor selection and dosing regimens may influence treatment outcomes, though this reflects real-world clinical practice patterns.

The clinical implications of our findings extend beyond the specific comparison of donafenib versus regorafenib. The framework of drug repositioning demonstrated in this study may serve as a model for optimizing treatment sequences with other underutilized first-line agents, including sorafenib and lenvatinib. As multiple effective first-line options become available, the strategic repositioning of these agents in second-line settings represents a rational approach to maximizing therapeutic potential while expanding treatment options for patients who progress on bevacizumab-based combinations.

Future research priorities should focus on prospective randomized trials comparing different second-line strategies following progression on various first-line regimens. The development of predictive biomarkers for treatment selection and investigation of optimal treatment sequences incorporating newer therapeutic modalities remain critical areas for advancement. Additionally, cost-effectiveness analyses of drug repositioning strategies may inform healthcare policy decisions and resource allocation.^[24]^

## 5. Conclusion

This study establishes donafenib-immunotherapy as a viable second-line alternative to regorafenib-based therapy following bevacizumab-immunotherapy progression in advanced HCC. Similar efficacy and safety outcomes, alongside drug repositioning rationale, support incorporating this strategy into clinical guidelines and treatment algorithms. These findings contribute to addressing the current disparity between abundant first-line options and limited second-line evidence, potentially optimizing treatment sequences in the era of combination immunotherapy for advanced HCC.

## Acknowledgments

We thank LetPub (www.letpub.com) for linguistic assistance and pre-submission expert review. This research was supported by the National Key Clinical Specialties Construction Program and the project “Effectiveness and Safety Analysis of CalliSpheres® Drug-Eluting Beads Loaded with Idarubicin Hydrochloride via Transarterial Chemoembolization for Hepatocellular Carcinoma” (Grant Number: YXJL-2022-0105-0020).

## Author contributions

**Data curation:** Xianyong Li, Xin Li, Yu Jiang, Qingyun Xie.

**Formal analysis:** Xiaoshuang Chen.

**Funding acquisition:** Ping Xie, Lei Cao.

**Investigation:** Xiaoshuang Chen.

**Methodology:** Xiaoshuang Chen.

**Project administration:** Ping Xie, Lei Cao.

**Software:** Xiaoshuang Chen, Xianyong Li, Xin Li, Yu Jiang, Qingyun Xie.

**Supervision:** Ping Xie, Lei Cao.

**Validation:** Xiaoshuang Chen.

**Visualization:** Xiaoshuang Chen.

**Writing – original draft:** Xiaoshuang Chen, Xianyong Li, Xin Li, Yu Jiang, Qingyun Xie.

**Writing – review & editing:** Ping Xie, Lei Cao.

## Supplementary Material



## References

[1] Fitzmaurice C Akinyemiju TF Al Lami FH. Global, regional, and national cancer incidence, mortality, years of life lost, years lived with disability, and disability-adjusted life-years for 29 cancer groups, 1990 to 2016: a systematic analysis for the global burden of disease study. JAMA Oncol. 2018;4:1553–68. 29860482 10.1001/jamaoncol.2018.2706 PMC6248091

[2] Ghouri YA Mian I Rowe JH. Review of hepatocellular carcinoma: epidemiology, etiology, and carcinogenesis. J Carcinog. 2017;16:1. 28694740 10.4103/jcar.JCar_9_16 PMC5490340

[3] Zhou J Sun H Wang Z. China liver cancer guidelines for the diagnosis and treatment of hepatocellular carcinoma (2024 Edition). Liver Cancer. 2025;14:779–835. 41063733 10.1159/000546574 PMC12503762

[4] Llovet JM Ricci S Mazzaferro V. Sorafenib in advanced hepatocellular carcinoma. N Engl J Med. 2008;359:378–90. 18650514 10.1056/NEJMoa0708857

[5] Finn RS Qin S Ikeda M. Atezolizumab plus bevacizumab in unresectable hepatocellular carcinoma. N Engl J Med. 2020;382:1894–905. 32402160 10.1056/NEJMoa1915745

[6] Ren Z Xu J Bai Y. Sintilimab plus a bevacizumab biosimilar (IBI305) versus sorafenib in unresectable hepatocellular carcinoma (ORIENT-32): a randomised, open-label, phase 2–3 study. Lancet Oncol. 2021;22:977–90. 34143971 10.1016/S1470-2045(21)00252-7

[7] Qin S Bi F Gu S. Donafenib versus sorafenib in first-line treatment of unresectable or metastatic hepatocellular carcinoma: a randomized, open-label, parallel-controlled phase II-III trial. J Clin Oncol. 2021;39:3002–11. 34185551 10.1200/JCO.21.00163 PMC8445562

[8] Bruix J Qin S Merle P. Regorafenib for patients with hepatocellular carcinoma who progressed on sorafenib treatment (RESORCE): a randomised, double-blind, placebo-controlled, phase 3 trial. Lancet. 2017;389:56–66. 27932229 10.1016/S0140-6736(16)32453-9

[9] Zhu AX Kang YK Yen CJ. Ramucirumab after sorafenib in patients with advanced hepatocellular carcinoma and increased alpha-fetoproteinconcentrations (REACH-2): a randomised, double-blind, placebo-controlled, phase 3 trial. Lancet Oncol. 2019;20:282–96. 30665869 10.1016/S1470-2045(18)30937-9

[10] Kelley RK Ryoo BY Merle P. Second-line cabozantinib after sorafenib treatment for advanced hepatocellular carcinoma: a subgroup analysis of the phase 3 CELESTIAL trial. ESMO Open. 2020;5:e000714. 32847838 10.1136/esmoopen-2020-000714 PMC7451459

[11] Donne R Lujambio A. The liver cancer immune microenvironment: therapeutic implications for hepatocellular carcinoma. Hepatology. 2023;77:1773–96. 35989535 10.1002/hep.32740 PMC9941399

[12] Mospan AR Morris HL Fried MW. Real‐world evidence in hepatocellular carcinoma. Liver Int. 2021;41:61–7. 34155788 10.1111/liv.14864

[13] Kudo M Finn RS Qin S. Lenvatinib versus sorafenib in first-line treatment of patients with unresectable hepatocellular carcinoma: a randomised phase 3 non-inferiority trial. Lancet. 2018;391:1163–73. 29433850 10.1016/S0140-6736(18)30207-1

[14] Cheng AL Kang YK Chen Z. Efficacy and safety of sorafenib in patients in the Asia-Pacific region with advanced hepatocellular carcinoma: a phase III randomised, double-blind, placebo-controlled trial. Lancet Oncol. 2009;10:25–34. 19095497 10.1016/S1470-2045(08)70285-7

[15] Liu J Li X Zhang H. Safety, pharmacokinetics and efficacy of donafenib in treating advanced hepatocellular carcinoma: report from a phase 1b trial. Pharmazie. 2019;74:688–93. 31739839 10.1691/ph.2019.9626

[16] Liang J Chen M Yan G. Donafenib activates the p53 signaling pathway in hepatocellular carcinoma, induces ferroptosis, and enhances cell apoptosis. Clin Exp Med. 2025;25:29. 39753901 10.1007/s10238-024-01550-6 PMC11698805

[17] Kubes P Jenne C. Immune responses in the liver. Annu Rev Immunol. 2018;36:247–77. 29328785 10.1146/annurev-immunol-051116-052415

[18] European Association for the Study of the Liver. EASL clinical practice guidelines: management of hepatocellular carcinoma. J Hepatol. 2018;69:182–236. 29628281 10.1016/j.jhep.2018.03.019

[19] Gordan JD Kennedy EB Abou-Alfa GK. Systemic therapy for advanced hepatocellular carcinoma: ASCO guideline. J Clin Oncol. 2020;38:4317–45. 33197225 10.1200/JCO.20.02672

[20] Vogel A Chan SL Dawson LA. Hepatocellular carcinoma: ESMO Clinical Practice Guideline for diagnosis, treatment and follow-up. Ann Oncol. 2025;36:491–506. 39986353 10.1016/j.annonc.2025.02.006

[21] Personeni N Pressiani T Santoro A Rimassa L. Regorafenib in hepatocellular carcinoma: latest evidence and clinical implications. Drugs Context. 2018;7:212533. 30002715 10.7573/dic.212533 PMC6038776

[22] Chen R Ielasi L di Carlo A Tovoli F. Donafenib in hepatocellular carcinoma. Drugs Today (Barc. 2023;59:83–90. 36811408 10.1358/dot.2023.59.2.3507751

[23] Lu F Zhao K Ye M. Efficacy and safety of second-line therapies for advanced hepatocellular carcinoma: a network meta-analysis of randomized controlled trials. BMC Cancer. 2024;24:1023. 39160484 10.1186/s12885-024-12780-y PMC11331808

[24] Sun KX Cao SS Shi FH. First-line treatments for advanced hepatocellular carcinoma: a network meta-analysis and cost-effectiveness analysis in China and the United States. Ther Adv Gastroenterol. 2022;15:17562848221140662. 36518883 10.1177/17562848221140662 PMC9742927

