# Supplementary material for: Repositioned donafenib versus standard regorafenib for second-line HCC treatment: A matched cohort study
Source: Medicine (Baltimore). 2026 Apr 24;105(17):e48470. doi: 10.1097/MD.0000000000048470 (PMC13124404; doi:10.1097/MD.0000000000048470)
Supplement: Supplementary file 1 [file medi-105-e48470-s001.pdf]

Supplementary Table 1. Subgroup Analysis of Progression-Free Survival

Subgroup Variable	Category	Dona+IO group (n)	Rego+IO group (n)	Dona+IO group, months (95% CI)	Rego+IO group Median PFS, months (95% CI)	HR (95% CI)	P value
Overall	-	25	25	9.3 (6.5-13.1)	7.1 (3.3-13.8)	1.114 (0.581-2.134)	0.745
Gender	Male	22	21	9.3 (6.5-13.1)	7.1 (NR)	1.100 (0.548-2.209)	0.789
	Female	3	4	13.3 (NR)	5.5 (NR)	1.029 (0.143-7.417)	0.977
Age	>60 years	7	8	12.2 (NR)	4.1 (NR)	1.573 (0.472-5.242)	0.461
	≤60 years	18	17	9.3 (NR)	7.1 (NR)	1.019 (0.458-2.267)	0.964
HBV infection	Yes	21	19	9.3 (NR)	5.5 (NR)	1.201 (0.575-2.507)	0.626
	No	4	6	9.1 (NR)	7.7 (NR)	1.224 (0.268-5.599)	0.794
HCV infection	No	25	22	9.3 (6.5-13.1)	5.5 (NR)	1.082 (0.552-2.121)	0.819
Liver cirrhosis	Yes	16	15	7.6 (NR)	7.7 (NR)	0.866 (0.361-2.075)	0.747
	No	9	10	9.9 (NR)	4.0 (NR)	1.594 (0.541-4.696)	0.398
BCLC staging	Stage B	7	6	7.0 (NR)	4.9 (NR)	1.331 (0.292-6.072)	0.712

	Stage C	18	19	9.4 (NR)	7.1 (3.3-13.8)	1.038 (0.491-2.195)	0.923
Child-Pugh score	A5	17	16	9.4 (NR)	5.4 (NR)	1.397 (0.630-3.096)	0.411
	B6	8	8	4.9 (NR)	7.7 (NR)	0.799 (0.225-2.839)	0.728
Ascites	Yes	9	5	10.3 (NR)	12.4 (NR)	0.590 (0.148-2.361)	0.456
	No	16	20	7.6 (NR)	5.3 (NR)	1.362 (0.623-2.977)	0.439
Tumor size (cm)	>5	19	13	7.6 (NR)	7.1 (NR)	0.881 (0.366-2.121)	0.778
	≤5	6	12	9.9 (NR)	5.3 (NR)	1.984 (0.600-6.566)	0.262
Tumor number	1	4	6	7.9 (NR)	8.6 (NR)	0.594 (0.132-2.683)	0.498
	2-3	11	13	7.6 (NR)	5.3 (NR)	1.772 (0.624-5.031)	0.283
	>3	10	6	9.3 (NR)	7.7 (NR)	0.849 (0.258-2.792)	0.787
Tumor morphology	Nodular	14	12	7.6 (NR)	7.7 (NR)	0.898 (0.369-2.184)	0.812
	Massive	11	13	9.9 (NR)	4.9 (NR)	1.304 (0.498-3.415)	0.589
PVTT	No	16	19	11.2 (NR)	7.7 (NR)	1.164 (0.527-2.570)	0.708
	Yes	9	6	9.3 (NR)	5.5 (NR)	0.990 (0.287-3.420)	0.988

Extrahepatic metastasis	No	19	17	9.4 (NR)	5.5 (NR)	1.563 (0.709-3.446)	0.268
	Yes	6	8	7.6 (NR)	7.1 (NR)	0.740 (0.210-2.605)	0.639
ECOG performance status	0	21	20	7.6 (NR)	5.4 (3.1-13.8)	1.101 (0.545-2.222)	0.789
	1	4	5	12.2 (NR)	7.1 (NR)	2.149 (0.191-24.138)	0.535
AFP (ng/mL)	≤400	18	19	9.3 (NR)	7.1 (NR)	0.975 (0.445-2.135)	0.949
	>400	7	6	7.0 (NR)	4.9 (NR)	1.659 (0.412-6.671)	0.476

Table Caption: Dona+IO group = Donafenib + immunotherapy group; Rego+IO group = Regorafenib + immunotherapy group; PFS = Progression-free survival; OS = Overall survival; HR = Hazard ratio; CI = Confidence interval; NR = Not reached; NE = Not estimable; HBV = Hepatitis B virus; HCV = Hepatitis C virus; BCLC = Barcelona Clinic Liver Cancer; ECOG = Eastern Cooperative Oncology Group; AFP = Alpha-fetoprotein; PVTT = Portal vein tumor thrombosis

Supplementary Table 2. Subgroup Analysis of Overall Survival

Subgroup Variable	Category	Dona+IO group (n)	Rego+IO group (n)	Dona+IO group, months (95% CI)	Rego+IO group Median PFS, months (95% CI)	HR (95% CI)	P value
Overall	-	25	25	25.8 (NR)	17.4 (NR)	1.517 (0.685-3.359)	0.304
Gender	Male	22	21	25.8 (NR)	19.2 (NR)	1.799 (0.735-4.404)	0.199
	Female	3	4	NR	14.7 (NR)	1.862 (0.187-18.531)	0.596
Age	>60 years	7	8	25.8 (NR)	32.3 (NR)	0.910 (0.197-4.203)	0.904
	≤60 years	18	17	NR	13.5 (NR)	1.562 (0.603-4.045)	0.358
HBV infection	Yes	21	19	25.8 (NR)	13.5 (NR)	2.096 (0.738-5.951)	0.165
	No	4	6	13.5 (NR)	14.4 (NR)	0.778 (0.206-2.940)	0.712
HCV infection	No	25	22	25.8 (NR)	13.5 (NR)	1.410 (0.619-3.211)	0.414
Liver cirrhosis	Yes	16	15	25.8 (NR)	17.4 (NR)	1.280 (0.474-3.457)	0.626
	No	9	10	21.4 (NR)	13.5 (NR)	4.057 (0.728-22.596)	0.11
BCLC staging	Stage B	7	6	21.4 (NR)	17.4 (NR)	4.386 (0.452-42.573)	0.202

	Stage C	18	19	25.8 (NR)	13.5 (NR)	1.400 (0.558-3.516)	0.473
Child-Pugh score	A5	17	16	21.4 (NR)	13.5 (NR)	1.265 (0.466-3.437)	0.644
	B6	8	8	29.9 (NR)	17.4 (NR)	1.555 (0.289-8.356)	0.607
Ascites	Yes	9	5	NR	17.4 (NR)	3.593 (0.688-18.772)	0.13
	No	16	20	21.4 (NR)	13.5 (NR)	0.958 (0.377-2.440)	0.929
Tumor size (cm)	>5	19	13	25.8 (NR)	13.5 (NR)	1.506 (0.539-4.212)	0.435
	≤5	6	12	21.4 (NR)	17.4 (NR)	1.178 (0.279-4.970)	0.824
Tumor number	1	4	6	NR	10.4 (NR)	NE	0.999
	2-3	11	13	25.8 (NR)	19.2 (NR)	0.808 (0.240-2.726)	0.732
	>3	10	6	16.3 (NR)	17.4 (NR)	0.590 (0.138-2.524)	0.477
Tumor morphology	Nodular	14	12	21.4 (NR)	23.8 (NR)	0.838 (0.287-2.445)	0.746
	Massive	11	13	NR	13.5 (NR)	3.794 (0.947-15.206)	0.06
PVTT	No	16	19	21.4 (NR)	17.4 (NR)	1.206 (0.452-3.218)	0.708
	Yes	9	6	25.8 (NR)	13.5 (NR)	3.465 (0.622-19.317)	0.156

Extrahepatic metastasis	No	19	17	25.8 (NR)	12.4 (NR)	3.820 (1.120-13.025)	0.032
	Yes	6	8	12.1 (NR)	23.8 (NR)	0.468 (0.103-2.131)	0.327
ECOG performance status	0	21	20	25.8 (NR)	19.2 (NR)	1.563 (0.645-3.787)	0.323
	1	4	5	7.2 (NR)	13.5 (NR)	1.269 (0.199-8.077)	0.801
AFP (ng/mL)	≤400	18	19	29.9 (NR)	13.5 (NR)	1.803 (0.646-5.032)	0.26
	>400	7	6	21.4 (NR)	17.4 (NR)	1.679 (0.372-7.586)	0.501

Table Caption: Dona+IO group = Donafenib + immunotherapy group; Rego+IO group = Regorafenib + immunotherapy group; PFS = Progression-free survival; OS = Overall survival; HR = Hazard ratio; CI = Confidence interval; NR = Not reached; NE = Not estimable; HBV = Hepatitis B virus; HCV = Hepatitis C virus; BCLC = Barcelona Clinic Liver Cancer; ECOG = Eastern Cooperative Oncology Group; AFP = Alpha-fetoprotein; PVTT = Portal vein tumor thrombosis
